# The Use of Mobile Health Applications to Improve Patient Experience: Cross-Sectional Study in Chinese Public Hospitals

**DOI:** 10.2196/mhealth.9145

**Published:** 2018-05-23

**Authors:** Chuntao Lu, Yinhuan Hu, Jinzhu Xie, Qiang Fu, Isabella Leigh, Samuel Governor, Guanping Wang

**Affiliations:** ^1^ School of Medicine and Health Management Tongji Medical College Huazhong University of Science and Technology Wuhan China; ^2^ Department of Epidemiology and Biostatistics College for Public Health and Social Justice Saint Louis University Missouri, MO United States; ^3^ Department of Communication Morrissey College of Arts and Sciences Boston College Chestnut Hill, MA United States; ^4^ Union Hospital Tongji Medical College Huazhong University of Science and Technology Wuhan China

**Keywords:** mobile applications, technology, outpatients, patient satisfaction, surveys and questionnaire

## Abstract

**Background:**

The proliferation of mobile health apps has greatly changed the way society accesses the health care industry. However, despite the widespread use of mobile health apps by patients in China, there has been little research that evaluates the effect of mobile health apps on patient experience during hospital visits.

**Objective:**

The purpose of our study was to examine whether the use of mobile health apps improves patient experience and to find out the difference in patient experience between users and nonusers and the characteristics associated with the users of these apps.

**Methods:**

We used the Chinese Outpatient Experience Questionnaire to survey patient experience. A sample of 300 outpatients was randomly selected from 3 comprehensive public hospitals (3 tertiary hospitals) in Hubei province, China. Each hospital randomly selected 50 respondents from mobile health app users and 50 from nonusers. A chi-square test was employed to compare the different categorical characteristics between mobile health app users and nonusers. A *t* test was used to test the significance in continuous variables between user scores and nonuser scores. Multiple linear regression was conducted to determine whether the use of mobile health apps during hospital visits was associated with patient experience.

**Results:**

The users and nonusers differed in age (χ^2^_2_=12.2, *P*=.002), education (χ^2^_3_=9.3, *P*=.03), living place (χ^2^_1_=7.7, *P*=.006), and the need for specialists (χ^2^_4_=11.0, *P*=.03). Compared with nonusers, mobile health app users in China were younger, better educated, living in urban areas, and had higher demands for specialists. In addition, mobile health app users gave significantly higher scores than nonusers in total patient experience scores (*t*_298_=3.919, *P*<.001), the 18 items and the 5 dimensions of physician-patient communication (*t*_298_=2.93, *P*=.004), health information (*t*_298_=3.556, *P*<.001), medical service fees (*t*_298_=3.991, *P*<.001), short-term outcome (*t*_298_=4.533, *P*<.001), and general satisfaction (*t*_298_=4.304, *P*<.001). Multiple linear regression results showed that the use of mobile health apps during hospital visits influenced patient experience (*t*_289_=3.143, *P*=.002). After controlling for other factors, it was shown that the use of mobile health apps increased the outpatient experience scores by 17.7%. Additional results from the study found that the self-rated health status (*t*_289_=3.746, *P*<.001) and monthly income of patients (*t*_289_=2.416, *P*=.02) influenced the patient experience as well.

**Conclusions:**

The use of mobile health apps could improve patient experience, especially with regard to accessing health information, making physician-patient communication more convenient, ensuring transparency in medical charge, and ameliorating short-term outcomes. All of these may contribute to positive health outcomes. Therefore, we should encourage the adoption of mobile health apps in health care settings so as to improve patient experience.

## Introduction

### Background

Patient experience is the overall satisfaction a patient gets during the course of receiving care or treatment. This satisfaction is viewed by patients subjectively or based on objective facts and is derived from the sum of their interactions with different factors at a health care setting that influence their perceptions about the quality of health care delivery at that setting [1,2]. Patient experience is an important outcome of medical care [3] and regarded as one of the central pillars of health care quality [4]. Policy makers worldwide increasingly prefer using patient-experience data rather than performance indicators to assess the quality of health care services [4]. Various patient experience questionnaires such as the Picker scale [5] and the Outpatient Experiences Questionnaire [6] have been developed in efforts to measure patient experience using different factors or dimensions at health care settings and the socioeconomic characteristics of patients. Patient experience is strongly correlated with the quality of health care delivery, which involves outcomes such as adherence to treatments, access to preventative care, and patient safety [7]. Qualitative studies have found that the more positive the experience of patients is in accessing care, facilities, and communication with physicians, the better their overall satisfaction about the quality of health care delivery [8].

In recent years, the emergence of mobile health apps in health care management has helped to overcome geographical and organizational barriers to improve health care delivery [9]. In 2018, about 50% of mobile phone users have at least 1 mobile health app on their mobile phones [10]. The Agency of Healthcare Research and Quality has developed an instrument to measure patient experience in relation to health information technology [11]. Evidence has confirmed that patient experience can be improved by mobile health apps through which reminders and diagnostic information are delivered to patients [12]. A series of research have found that mobile health apps can improve adherence to medication for patients with chronic diseases [13], monitor diet behaviors for patients with diabetes, and encourage the collection of blood pressure readings for hypertensive patients [14].

### Objectives

A number of Chinese hospitals have begun applying mobile health apps to improve health care services. These mobile health apps can provide information for many services such as hospital guidance, health care consultancy, visiting appointment and registration, medical result checking, medical charge payment, and inquiry [15]. Patients can freely download a hospital’s mobile health app by searching for the app in application stores or scanning quick response codes (two-dimensional codes) available in the hospital and on the hospital website [16]. Those mobile health apps have allowed for more efficient responses to patient demands and reduced the amount of time a patient spends in long queues trying to access care in hospital settings. Thus, mobile health apps play a positive role in improving the efficiency and quality of health care delivery [17]. Despite this surge in the use of mobile health apps in China, evidence to evaluate their effectiveness in improving health care delivery is lacking. Therefore, the purpose of our study was to examine whether the use of mobile health apps improves patient experience and to compare the patient experience between users and nonusers and the characteristics associated with the users of these apps.

## Methods

### Questionnaire Design

The Chinese Outpatient Experience Questionnaire was the basis of our survey, which included 6 dimensions (physical environment and convenience, physician-patient communication, health information, medical service fees, short-term outcome, and general satisfaction), 28 items, and patient characteristics (gender, age, education, marital status, monthly income, payment, living place, specialty, self-rated health status) with good reliability and validity [18]. In the questionnaire, we added another question in the characteristics section— “Did you use mobile health apps during this visit?”—to divide the users and nonusers.

### Sample and Procedure

The survey was carried out in August 2016. A sample of 300 respondents was randomly selected from 3 comprehensive public hospitals (3 tertiary hospitals) in Hubei province, China. We adopted the following inclusion criteria for respondents: aged ≥18 years; completed visit procedure before leaving hospital; consented to participate in the study; and offered their own experience about the visit accurately and independently. We chose 3 hospitals that provided similar mobile health apps (ie, displaying information about hospitals and physicians, communicating with physicians, providing hospital appointment and registration, checking medical results, monitoring status of queues, and paying for medical service fees), had a similar number of outpatients per year, and had full-fledged mobile health apps suited for this survey. Trained interviewers randomly selected respondents who met the inclusion criteria and conducted the face-to-face interviews. If the respondents refused to answer any question, then the questionnaire for that respondent was deleted. After a questionnaire was completed, the interviewer reviewed it to ensure that no errors were made. This was done until each hospital had surveyed 50 mobile health app users and 50 nonusers. Every participant provided a score based on their visit experience. The scores represented not only the patients’ evaluation of the health services provided to them but also the extent to which patient experience could be improved.

### Measures

In multiple linear regression, the dependent variable was the total patient experience score. We used the Chinese Outpatient Experience Questionnaire to calculate patient experience score. The response to each of the 28 items was given based on a 5-point Likert scale, with 5 representing the best experience and 1 representing the worst. For example, “What do you think of the waiting time in the hospital?” (coded as 1=very long, 5=very short). Each dimension score was calculated by summing all item scores in the dimension and then dividing that sum by the number of items in that dimension. The total patient experience score was the total questionnaire score, which was calculated by summing the scores of the 28 items in the questionnaire and then dividing that sum by the total number of items. The total patient experience score ranged from 1 to 5.

To determine possible areas for improvement, we normalized each respondent item’s score to 0-100 by using the following formula: Normalized score=100×(Respondent’s selected response value−Minimum response value on scale)/(Maximum response value−Minimum response value) [19]. We determined that the distance between a patient experience score and 100 is the gap that must be improved.

The independent variables included the 9 characteristics and whether mobile health apps were used during a visit (coded as 1=yes, 0=no). The 9 characteristics are presented in Table 1. Education, payment, specialty, and self-rated health status were recoded as dummy variables and set the first indicator as the reference.

### Statistical Analysis

Data entry and management were performed using Epidata 3.1 (“The EpiData Association” Odense, Denmark) Double-entry data input was used to ensure accuracy. A chi-square test was employed to compare the different categorical characteristics between mobile health app users and nonusers. A *t* test was used to test the mean difference in patient experience scores between mobile health app users and nonusers. Multiple linear regression was conducted to determine whether the use of mobile health apps during the hospital visit was associated with patient experience. A cutoff of *P*<.05 (in a 2-tailed test) was used to determine the statistical significance for all tests. All analyses were conducted using SPSS Version 20.0, 2011 (IBM SPSS, Inc, Chicago, IL).

## Results

### Patient Characteristics of Mobile Health Application Users and Nonusers

In the 300 samples, the mean age was 33 years (SD 0.902). Females accounted for 56.3% (169/300), and 56.0% (168/300) of respondents had a college or higher education. Respondents living in urban areas accounted for 74.3% (223/300), and 88.7% (266/300) of respondents had a monthly income more than ￥2000 (the per capita annual disposable income of China was ￥23,821 in 2016 [20]). We combined the category of divorced/widowed/separated, which had a small number of respondents, into the married category, therefore, ever married was 78.0% (234/300). A total of 66.7% (200/300) of respondents paid completely out of pocket for medical service fees. For self-rated health status, 47.0% (141/300) of individuals rated their health as good or better. The 4 major medical specialties—internal medicine, surgery, obstetrics and gynecology, and pediatrics—were the most common services requested by outpatients, which accounted for 75.7% (227/300) of respondents.

The characteristics distributions are presented in Table 1; a chi-square test was used to examine the difference in patients’ characteristics. The mobile health app users and nonusers differed in age (χ^2^_2_=12.2, *P*=.002), education (χ^2^_3_=9.3, *P*=.03), living place (χ^2^_1_=7.7, *P*=.006), and their request for specialty services (χ^2^_4_=11.0, *P*=.03). Compared with nonusers, the mobile health app users were younger, better educated, lived in urban areas, and had more requests for medical specialists.

### Differences in Patient Experience Between Mobile Health Application Users and Nonusers

Patient experience scores of mobile health app users and nonusers are shown in Table 2. The *t* test results showed that there was a significant difference in total patient experience scores, the 5 dimensions, and the 18 items between the 2 groups.

In total patient experience scores, mobile health app users gave significantly higher scores than nonusers (*t*_298_=3.919, *P*<.001). In the dimensions of physician-patient communication (*t*_298_=2.93, *P*=.004), health information (*t*_298_=3.556, *P*<.001), medical service fees (*t*_298_=3.991, *P*<.001), short-term outcome (*t*_298_=4.533, *P*<.001), and general satisfaction (*t*_298_=4.304, *P*<.001), mobile health app users obtained significantly higher scores than nonusers as well. The same relationship was observed in the 18 items, as the app users obtained significantly higher scores than their nonuser counterparts (Table 2).

Although the dimension of physical environment and convenience was not significantly different between the 2 groups (*t*_298_=1.285, *P*=.20), mobile health app users obtained significantly higher scores in the item concerning convenience of the registration procedure, indicating that the apps played a positive role in registering for doctor appointments.

**Table 1:** Difference in respondents’ characteristics of mobile health app users and nonusers.

Characteristic		App users, n (%)	Nonusers, n (%)	Chi-square (*df*)	*P* value
**Gender**				0.1 (1)	.42
	Male	62 (41.3)	69 (46.0)		
	Female	88 (58.7)	81 (54.0)		
**Age**				12.2 (2)	.002^a^
	≤44	124 (82.7)	98 (65.3)		
	45-64	19 (12.7)	42 (28.0)		
	≥65	7 (4.6)	10 (6.7)		
**Education**				9.3 (3)	.03^a^
	Elementary and below	3 (2.0)	12 (8.0)		
	Middle school	15 (10.0)	22 (14.7)		
	High school	38 (25.3)	42 (28.0)		
	College or above	94 (62.7)	74 (49.3)		
**Monthly income (￥)**				8.3 (4)	.08
	≤1999	16 (10.7)	18 (12.0)		
	2000-2999	25 (16.7)	42 (28.0)		
	3000-3999	35 (23.3)	34 (22.7)		
	4000-4999	28 (18.7)	27 (18.0)		
	≥5000	46 (30.6)	29 (19.3)		
**Marital status**				0.1 (1)	.78
	Single	34 (22.7)	32 (21.3)		
	Ever married	116 (77.3)	118 (78.7)		
**Living place**				7.7 (1)	.006^a^
	Urban areas	122 (81.3)	101 (67.3)		
	Rural areas	28 (18.7)	49 (32.7)		
**Payment**				1.3 (2)	.52
	Pay completely out of pocket	104 (69.3)	96 (64.0)		
	Partial reimbursement	40 (26.7)	49 (32.7)		
	Complete reimbursement	6 (4.0)	5 (3.3)		
**Specialty services**				11.0 (4)	.03^a^
	Internal medicine	41 (27.3)	49 (32.7)		
	Surgery	37 (24.7)	42 (28.0)		
	Obstetrics and gynecology	29 (19.3)	10 (6.7)		
	Pediatrics	10 (6.7)	9 (6.0)		
	Others	33 (22.0)	40 (26.6)		
**Self-rated health status**				6.4 (3)	.09
	Poor	8 (5.3)	16 (10.7)		
	Fair	62 (41.3)	73 (48.7)		
	Good	64 (42.7)	51 (34.0)		
	Very good	16 (10.7)	10 (6.7)		

^a^Represents the significance between the 2 groups.

**Table 2:** Patient experience scores of mobile health app users and nonusers.

Dimension/item		App users scores *x̄* (S)	Nonusers scores scores *x̄* (S)	*t* test (*df*)	*P* value
**Physical environment and convenience**		3.50 (0.54)	3.43 (0.51)	1.285 (298)	.20^c^
	Waiting time	2.37 (0.79)	2.41 (0.85)	−0.494 (298)	.62
	Easy registration procedure	3.97 (0.89)	3.67 (0.92)	2.808 (298)	.005^a^
	Convenient dispensary	3.89 (0.96)	3.69 (0.84)	1.913 (298)	.06
	Clear signs	4.05 (0.78)	3.94 (0.85)	1.130 (298)	.26
	Clean clinics	4.04 (0.74)	4.02 (0.70)	0.240 (298)	.81
	Quiet clinics	2.71 (1.04)	2.81 (0.96)	−0.924 (298)	.36
**Physician-patient communication**		3.73 (0.59)	3.52 (0.64)	2.930 (298)	.004^a,c^
	Clear explanation	4.20 (0.62)	3.96 (0.72)	3.079 (298)	.002^a^
	Careful listening	4.02 (0.79)	3.85 (0.87)	1.804 (298)	.07
	Enough time for communication	3.28 (0.96)	3.21 (0.99)	0.594 (298)	.55
	Courtesy and respect attitude	3.95 (0.78)	3.82 (0.79)	1.474 (298)	.14
	Cared about anxieties or fears	3.49 (0.90)	3.28 (0.90)	1.987 (298)	.048^a^
	Involve in decision making	3.15 (1.02)	2.94 (1.01)	1.768 (298)	.08
	Respect opinions	3.76 (0.76)	3.40 (0.88)	3.812 (298)	<.001^a,b^
	Protect personal privacy	3.97 (0.80)	3.69 (0.95)	2.766 (298)	.006^a,b^
**Health information**		3.91 (0.67)	3.61 (0.80)	3.556 (298)	<.001^a,b,c^
	Explanations for your illness	4.13 (0.77)	3.86 (0.87)	2.894 (298)	.004^a^
	Dangerous signals at home	4.03 (0.82)	3.74 (1.06)	2.624 (298)	.009^a,b^
	Health knowledge	3.74 (0.90)	3.51 (1.16)	1.949 (298)	.05^b^
	Explain following examination	3.93 (0.89)	3.51 (1.11)	3.615 (298)	<.001^a,b^
	Explain examination result	3.93 (0.94)	3.61 (0.93)	3.034 (298)	.003^a^
	Explain drug effects in a way you could understand	3.70 (0.89)	3.37 (1.02)	3.020 (298)	.003^a,b^
	Medication precautions	3.93 (0.82)	3.68 (1.07)	2.305 (298)	.02^a,b^
**Medical service fees**		3.52 (0.67)	3.19 (0.77)	3.991 (298)	<.001^a,c^
	Reasonable charge	3.43 (0.68)	3.05 (0.90)	4.117 (298)	<.001^a^
	Transparent charge	3.61 (0.90)	3.35 (0.94)	2.504 (298)	.01^a^
	Affordable charge	3.53 (0.88)	3.18 (0.86)	3.518 (298)	<.001^a^
**Short-term outcome**		3.90 (0.63)	3.53 (0.77)	4.533 (298)	<.001^a,b,c^
	Reduce/prevent from health problems	4.03 (0.70)	3.63 (0.77)	4.703 (298)	<.001^a,b^
	Handle health problems after visit	3.76 (0.73)	3.43 (0.96)	3.390 (298)	<.001^a,b^
**General satisfaction**		3.93 (0.72)	3.55 (0.82)	4.304 (298)	<.001^a,b,c^
	Satisfaction overall	3.80 (0.74)	3.43 (0.87)	3.896 (298)	<.001^a,b^
	Choose this hospital again	4.07 (0.80)	3.67 (0.88)	4.117 (298)	<.001^a^
Total patient experience scores		3.72 (0.50)	3.49 (0.56)	3.919 (298)	<.001^a^

^a^Represents a significant difference between 2 groups.

^b^Refers to *t* test.

^c^Represents the dimensions in the questionnaire.

**Table 3:** Factors associated with patient experience scores in the multiple linear regression.

Variables	*β*	SE	*t* test (*df*)	*P* value
Constant	2.987	0.169	17.681 (289)	<.001^a^
Whether app was used	.193	0.062	3.143 (289)	.002^a^
Monthly income	.061	0.025	2.416 (289)	.02^a^
Safe-rated health 1	.036	0.116	0.31 (289)	.76
Self-rated health 2	.120	0.118	1.01 (289)	.31
Self-rated health 3	.561	0.15	3.746 (289)	<.001^a^

^a^Represents the variable is significant in the multiple linear regression.

### Influence of Mobile Health Application on Patient Experience

Table 3 presents the results derived from multiple linear regression. The initial model included all independent variables, and the final model included only 3 significant variables. Whether mobile health apps were used or not during the hospital visit was a significant predictor that influenced patient experience scores (*t*_289_=3.143, *P*=.002). The standardized coefficient of whether app was used was .177; thus, when other covariates were held constant, the use of the mobile health apps increased the patient experience scores by 17.7%. Monthly income (*t*_289_=2.416, *P*=.02) was a significant factor that influenced the patient experience scores as well. Meanwhile, the self-rated health status was a factor that influenced patient experience. Patients who rated their health status better, would more likely to have better patient experience (*t*_289_=3.746, *P*<.001). Patients who used mobile health apps, those who had higher monthly income, and those who rated their health better were more likely to have a better experience during hospital visit. The model was fitted well to the data (*F*_10,289_=4.193, *P*<.001). We checked for collinearity by calculating the value of variance inflation factor (VIF). The VIF was between 1.095 and 6.222. As it is <10, there was no collinearity.

## Discussion

### Principal Findings

We found that the use of mobile health apps improved the overall patient experience. In our study, mobile health app users had a better experience in physician-patient communication, access to health information, payment of medical service fees, short-term outcomes, and general satisfaction. We also found that the mobile health app users were younger, better educated, lived in urban areas, and had higher requests for specialists.

The use of mobile health apps can save patients’ time throughout their visits. After choosing a medical specialist for an appointment, the patients can monitor queues on mobile health apps and arrive at the hospital just before their turn for their appointment. Patients can also use mobile health apps to pay for their medical service fees without queuing up for payment after finishing their medical tests and diagnostic procedures. Furthermore, mobile health apps can make medical service fees more transparent [21] by listing the items of medical services for which the patients are billed.

The relationship between physicians and patients in terms of communication has not been at its best in China [22]. Mobile technology can help improve the patient-physician relationship by allowing individuals and health care providers to establish a more effective communication channel [23]. With the emergence of mobile health apps, patients can communicate with physicians before and after their medical appointments through the mobile health apps to obtain medical consultations and other information about their health problems. Studies have confirmed that patient involvement in decision-making processes and effective communication are strongly associated with better self-reported clinical outcomes [24]. Considering previous studies, the effects of self-rated health status and monthly income on the mobile health app users found in our study were similar to the results reported by Bjertnaes [25]. However, as opposed to Esan’s research about the influential factors of patient experience [26], our study showed that age and type of payment were less significant in influencing patient experience.

There is increasing evidence that improved health care system delivery improves patient experience [27], which in turn results in better health outcomes for patients [7]. In our study, the overall patient experience was improved when the mobile health apps were used. Thus, we believe that the increased use of mobile health apps in health care settings could contribute to better health care outcomes.

Age, education, living place, and the requests for specialty-related services were associated with mobile health apps in our study, consistent with findings from Carroll and Ernsting [28,29]. Only 4.7% (7/150) of our surveyed users were elderly, which is similar to 5.2% of Chinese Internet users who are aged above 60 years [30]. It is reported that the biggest demographic change in the next 30 years will be the aging population, which will contribute to a dramatically increased incidence of diseases and lead to significantly higher demands for medical services [31]. The developers of mobile health apps should make the apps easy-to-use for the elderly population. Meanwhile, the broadband network covers 95% of administrative villages in China; the Internet users in rural areas have more tendency to use mobile phones to access the Internet [30]. Thus, health care service providers must encourage persons living in rural areas and those with lower educational attainment to use mobile health apps to access health care, as more people will have mobile phones with Internet access [28]. This will decrease the digital gap in the Chinese population [32].

### Limitations

There are some limitations in this study. First, because of the fluidity of the outpatients and the lack of their contact information in Chinese hospitals, we could not survey participants using a rigorous random sampling technique, which can result in selection bias. Second, patients can freely choose to use or not use the mobile health apps. Those who choose to use mobile health apps may have a more positive attitude toward technology than the nonusers. This potential difference could serve as a confounder in masking the patient experience. Third, the experiences of patients with chronic diseases who have to regularly return to the hospital may be different from those patients with acute diseases. However, we did not consider this factor in our survey.

### Conclusions

Mobile health app users in China are individuals who were younger, better educated, living in urban areas, and have more requests for specialists. Most importantly, our research provides evidence supporting the use of mobile health apps for improving patient experience. Mobile health apps display more accurate health information and transparent medical charge information. They also provide patients with more opportunities to communicate with physicians and may improve the relationship between physicians and patients. They also ameliorate short-term outcomes and increase general satisfaction of patients. All of these will improve patient experience and may contribute to positive health outcomes. Thus, the use of mobile health apps has great clinical significance. To improve the quality of health care delivery, we should encourage patients to adopt mobile health apps during their hospital visits, and hospitals should take advantage of mobile health apps to improve patient experience during hospital visits.

## Abbreviations

VIF: variance inflation factor
